# Understanding academic burnout in ethnic minority vocational students: a mixed-methods examination of career indecision, irrational beliefs, and academic self-efficacy

**DOI:** 10.3389/fpsyg.2026.1923573

**Published:** 2026-08-19

**Authors:** Liangfang Wu, Qi Wei, Zhu Yao, Lingling Fan

**Affiliations:** 1 College of Journalism and Communication, Hunan Normal University, Changsha, China; 2 School of Foreign Languages, Guangdong Polytechnic Normal University, Guangzhou, China; 3 Faculty of Arts and Education, The University of Auckland, Auckland, New Zealand

**Keywords:** academic burnout, academic self-efficacy, career indecision, ethnic minority students, irrational beliefs, secondary vocational education

## Abstract

**Introduction:**

Academic burnout represents an important concern among ethnic minority secondary vocational students, who may encounter distinctive challenges in career development and academic adaptation. However, the psychological processes linking career indecision to academic burnout remain insufficiently understood in this population. Guided by ABC theory and self-efficacy theory, this study examined the relationships among career indecision, irrational beliefs, academic self-efficacy, and academic burnout and explored how students' experiences contextualized these relationships.

**Methods:**

A sequential explanatory mixed-methods design was employed. Quantitative data were collected from 398 ethnic minority secondary vocational students in China and analyzed using structural equation modeling and bootstrap analyses. Subsequently, ten students were selected for semi-structured interviews, and the interview data were analyzed using thematic analysis.

**Results:**

Career indecision was positively associated with irrational beliefs (β = 0.32, *p* < 0.001) and negatively associated with academic self-efficacy (β = −0.39, *p* < 0.001). Significant indirect effects were identified through both the career indecision-irrational beliefs-academic burnout pathway and the career indecision-academic self-efficacy-academic burnout pathway. However, the hypothesized sequential indirect pathway involving both irrational beliefs and academic self-efficacy was not supported. The qualitative findings revealed fragmented career aspirations, uncertainty about future planning, and experiences of academic burnout associated with perceived major mismatch. Students also described limited access to career information, educational support, and opportunities for occupational exploration. These accounts helped contextualize how irrational beliefs and reduced academic self-efficacy could accompany career indecision and academic burnout.

**Discussion:**

The findings suggest that career indecision was associated with academic burnout through two distinct psychological pathways involving irrational beliefs and academic self-efficacy, rather than through the proposed sequential process. By integrating statistical relationships with students' contextual experiences, this study extends understanding of academic burnout among an understudied vocational student population. The findings also indicate the potential value of strengthening career guidance, occupational exploration, and academic support for ethnic minority secondary vocational students.

## Introduction

Academic burnout became a widespread concern in vocational education because of its detrimental effects on students' psychological wellbeing, academic performance, and long-term career development (Alsararatee et al., 2025). Previous studies had shown that academic burnout was associated with emotional exhaustion, learning disengagement, reduced motivation, poor academic achievement, and increased risk of school dropout (Yusof et al., 2023; Kovan, 2026). Existing research had identified multiple factors at the individual, school, family, and societal levels, including weak academic foundations, inadequate career guidance, low parental expectations, and negative stereotypes toward vocational education (Zhang et al., 2024; Bai et al., 2025).

These challenges might be particularly salient among ethnic minority secondary vocational students. Previous studies suggested that educational opportunities in China remained unevenly distributed across social and demographic groups despite the expansion of the education system (Guo et al., 2020). Ethnic minority students often faced additional developmental challenges related to educational access, family socioeconomic conditions, and career exploration opportunities, which might increase their vulnerability to career uncertainty and academic maladjustment (Doan et al., 2022; Yang et al., 2019).

Although previous research identified numerous predictors of academic burnout, several important gaps remained. First, existing studies primarily focused on general secondary school or university students, whereas ethnic minority secondary vocational students remained substantially underrepresented in the literature. Compared with mainstream student populations, these students often experienced distinctive educational, cultural, and career-development challenges that might shape their psychological adjustment and learning experiences differently. Second, previous studies largely examined isolated predictors of academic burnout, such as academic stress, learning motivation, or family support, without considering the systematic psychological processes through which career-related cognition might influence burnout. Third, although career indecision was associated with various adverse educational outcomes, relatively little was known about how career indecision contributed to academic burnout through multiple psychological mechanisms among ethnic minority secondary vocational students. Consequently, the psychological pathways linking career indecision and academic burnout remained insufficiently understood in this unique educational context.

**Figure 1:**
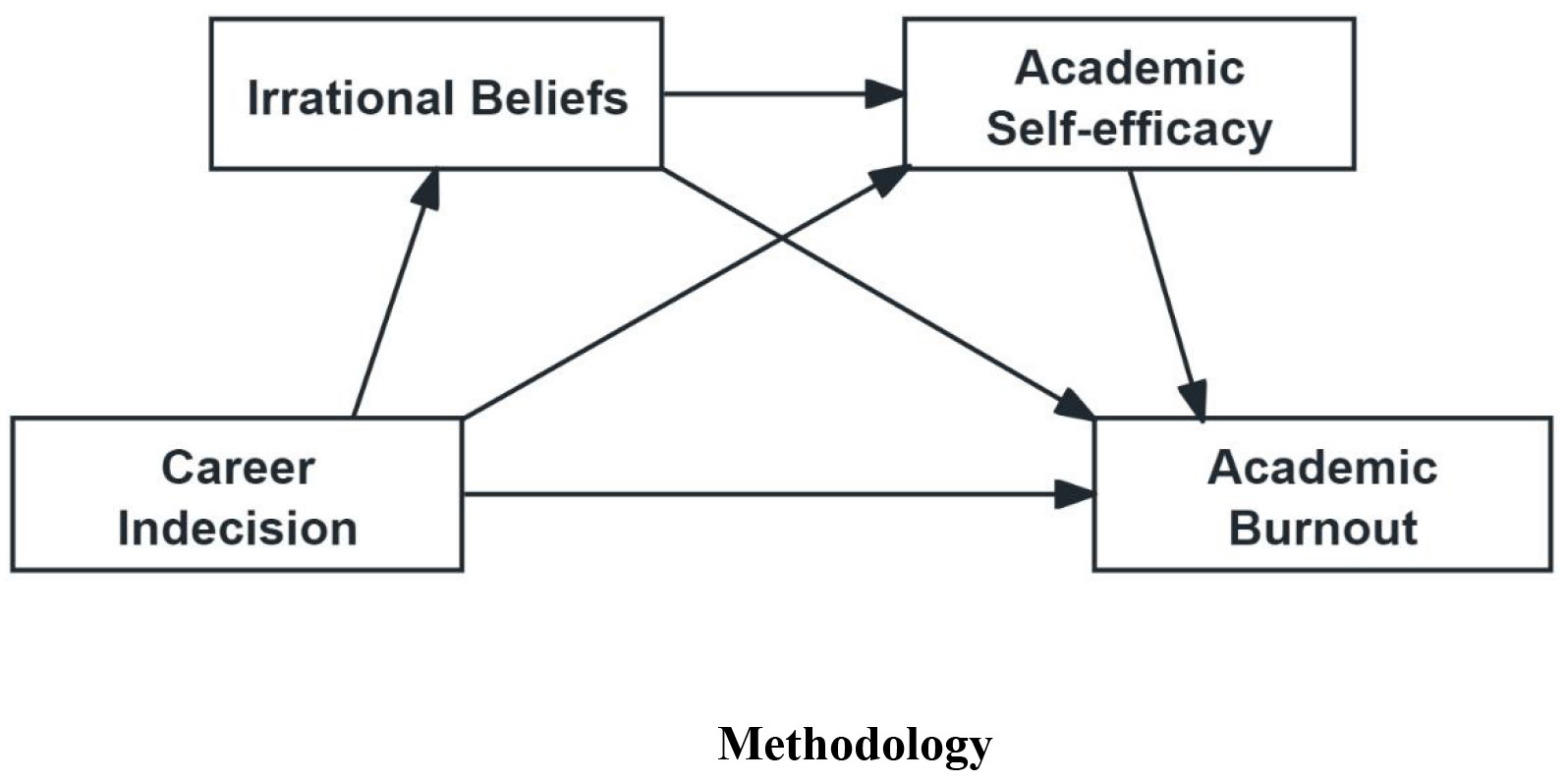
The hypothesized model.

To address these gaps, the present study drew upon ABC theory of emotion and self-efficacy theory to develop a theoretical framework explaining the psychological mechanisms underlying academic burnout. According to ABC theory, career indecision might function as a career-related stressor that elicits irrational beliefs regarding future occupations and personal competence, thereby increasing students‘ psychological vulnerability. Self-efficacy theory further suggested that these maladaptive beliefs might undermine students' confidence in managing academic demands, ultimately increasing their susceptibility to academic burnout. Integrating these two theoretical perspectives provided a coherent explanation for the proposed relationships among career indecision, irrational beliefs, academic self-efficacy, and academic burnout. Building upon this framework, the present study examined both the independent and sequential mediating roles of irrational beliefs and academic self-efficacy. Furthermore, by focusing on ethnic minority secondary vocational students—an understudied population, and adopting a sequential explanatory mixed-methods design that integrated quantitative and qualitative evidence, this study aimed to provide a more comprehensive understanding of the psychological mechanisms underlying academic burnout. The hypothesized research model is presented in Figure 1, and the following research questions and hypotheses were proposed.

### Career indecision and academic burnout

Career indecision (CI) was commonly assessed using the Career Decision Difficulties Questionnaire (CDDQ) (Gati and Saka, 2001), which was widely used to evaluate students' uncertainty and difficulties in career decision-making. Existing studies suggested that career-related uncertainty might negatively associate with psychological adjustment and academic functioning. Adolescents with higher levels of career indecision tended to report greater emotional distress and poorer academic outcomes (Amaral et al., 2023; Floris et al., 2024). Moreover, career uncertainty was associated with lower educational engagement (Datu and Buenconsejo, 2021) and reduced quality of life (Kusumawardani and Hidayat, 2024).

Academic burnout (AB), commonly measured using instruments such as the Maslach Burnout Inventory-Student Survey (MBI-SS) (Wu et al., 2010) was linked to multiple psychological and environmental factors. Among Chinese vocational students, external factors such as negative social media exposure were shown to exacerbate learning burnout (Zhang et al., 2024). However, such studies primarily identified external risk factors without sufficiently showing the underlying psychological mechanisms.

Several studies were directly examined the relationship between CI and AB. Kusumawardani and Hidayat (2024) reported that adolescents experiencing greater career indecision tended to exhibit higher levels of academic burnout, suggesting that uncertainty regarding future occupational direction may increase psychological pressure and learning disengagement. However, findings remained inconsistent across cultural contexts, and evidence involving ethnic minority secondary vocational students remains scarce.

Although previous studies suggested that career indecision might increase academic burnout, the psychological processes through which career indecision translated into burnout remained unclear. ABC theory suggested that maladaptive beliefs might represent an important cognitive mechanism linking career-related uncertainty with emotional maladjustment (Ellis, 1991).

### Career indecision and irrational beliefs

Irrational beliefs (IB), derived from Ellis's ABC theoretical framework, were widely examined as cognitive factors influencing emotional and behavioral outcomes. Previous studies showed that irrational beliefs frequently functioned as cognitive mechanisms linking external experiences with psychological consequences (Hua et al., 2022). In educational contexts, they might show students' stress responses and behavioral adjustment (Pascal and Chivu, 2022).

According to ABC theory, career indecision might function as an activating event that triggers maladaptive cognitive evaluations. Persistent uncertainty regarding future educational or occupational choices might foster irrational beliefs, such as excessive worry, perfectionistic expectations, and negative self-evaluation, thereby increasing psychological vulnerability. Empirical studies indicated that irrational occupational beliefs were significantly associated with career indecisiveness rather than career maturity (Hamamci and Esen Çoban, 2007). Similarly, adolescents experiencing difficulties in career planning were more likely to report stronger irrational occupational beliefs, while parental expectations and criticism also positively predicted career decision-making difficulties (Ari and Eşkisu, 2025). Evidence regarding the relationship between career indecision and irrational beliefs among ethnic minority secondary vocational students remained limited. Therefore, the present study hypothesized that career indecision would be positively associated with irrational beliefs.

### Career indecision and academic self-efficacy

Academic self-efficacy (AS) reflected students' confidence in their ability to complete academic tasks and achieve learning goals and is commonly measured using scales developed by Chemers et al. (2001). Existing studies suggest that career-related uncertainty is negatively associated with students' confidence and motivational beliefs. Students lacking clear future goals often exhibited weaker motivation and lower confidence regarding academic achievement. Starica (2012) found that academic self-efficacy and self-esteem significantly linked to career planning and career indecision. Similarly, Sidiropoulou-Dimakakou et al. (2012) reported that dysfunctional career thoughts increased decision-making difficulties, whereas self-efficacy acted as a protective factor.

More recently, Kholifah et al. (2025) demonstrated that self-efficacy served as a key mediating mechanism linking vocational students' career decision-making with academic adjustment. These findings suggested that self-efficacy might be shaped by external cognitive resources and career-related information. However, research examining the relationship between career indecision and academic self-efficacy among ethnic minority vocational students remained limited. This study hypothesized that career indecision was negatively associated with academic self-efficacy.

### Irrational beliefs and academic self-efficacy

Previous studies suggested that irrational beliefs might negatively relate to self-efficacy by distorting individuals' interpretations of challenges and failures. According to ABC theory, irrational beliefs distorted individuals' interpretations of academic experiences and increased negative evaluations of their own abilities. From the perspective of self-efficacy theory, these maladaptive cognitions were likely to undermine students' confidence in managing academic demands, thereby weakening academic self-efficacy.

Empirical evidence generally supported this relationship. Ozer and Akgun (2015) found that irrational beliefs negatively predicted students' academic self-efficacy. Similarly, Kabasakal and Emiroglu (2020) reported a negative association between irrational beliefs and adolescents' self-efficacy. Coşa and Cernat (2025) further emphasized the importance of irrational beliefs and self-efficacy in predicting academic performance. Although existing findings generally indicated that irrational beliefs might weaken self-efficacy beliefs, evidence involving ethnic minority vocational students remained scarce. This study hypothesized that irrational beliefs were negatively associated with academic self-efficacy.

### Irrational beliefs and academic burnout

Previous studies consistently suggested that maladaptive cognitive beliefs increased vulnerability to burnout by intensifying stress perception and emotional exhaustion. According to the ABC framework, irrational beliefs functioned as cognitive mechanisms linking activating events and emotional consequences (Ellis, 1991).

However, existing empirical evidence mainly came from teacher populations rather than student samples. Huk et al. (2019) reported that irrational beliefs positively predicted teacher burnout, while Gkontelos et al. (2023) similarly found that irrational beliefs exacerbated burnout symptoms.

Although these studies suggested that irrational beliefs might increase burnout risk, direct evidence among student populations, particularly ethnic minority secondary vocational students remained insufficient. Accordingly, the present study hypothesized that irrational beliefs would mediate the relationship between career indecision and academic burnout.

### Academic self-efficacy and academic burnout

Academic self-efficacy was consistently identified as an important protective factor against academic burnout across educational contexts. Previous studies among university students and nursing students found that stronger academic self-efficacy significantly predicted lower levels of burnout (Kong et al., 2021; Ma, 2024). Cong et al. (2024) further demonstrated that learning engagement partially mediated the relationship between academic self-efficacy and academic burnout. Similarly, Gao (2023) found that academic self-efficacy weakened the association between academic stress and burnout among Chinese adolescents. Özhan (2021) also identified self-efficacy as a protective factor through resilience mechanisms.

Bandura's self-efficacy theory suggested that students perceiving themselves as capable of managing academic demands were more likely to maintain engagement and cope effectively with learning challenges (Bandura, 1977). Nevertheless, evidence involving ethnic minority secondary vocational students remained limited. Therefore, the present study hypothesized that academic self-efficacy would be negatively associated with academic burnout.

### Parallel and sequential mediation

ABC theory suggested that career indecision might trigger irrational beliefs, whereas self-efficacy theory suggested that maladaptive beliefs might undermine students' confidence in managing academic demands. Together, these theoretical perspectives indicated that irrational beliefs and academic self-efficacy might operate as both independent and sequential mediators linking career indecision to academic burnout. However, this integrated psychological mechanism had received limited empirical attention, particularly among ethnic minority secondary vocational students.

To examine these proposed psychological relationships, the present study employed a sequential explanatory mixed-methods design. The hypothesized research model was presented in Figure 1. Based on this conceptual framework, the following research questions and hypotheses were proposed.

RQ1: Does career indecision predict academic burnout among ethnic minority secondary vocational students?

RQ2: Do irrational belief and academic self-efficacy mediate the relationship between career indecision and academic burnout?

RQ3: How do the qualitative findings reveal the psychological mechanisms underlying these relationships?

H1. Career indecision is positively associated with academic burnout.

H2. Career indecision is positively associated with irrational beliefs, whereas irrational beliefs are negatively associated with academic self-efficacy, which in turn is negatively associated with academic burnout.

H3. Irrational beliefs and academic self-efficacy mediate the relationship between career indecision and academic burnout, independently and sequentially.

## Methodology

### Research design

This study adopted a sequential explanatory mixed-methods design, consisting of a quantitative survey followed by a qualitative interview study. The quantitative phase was conducted first to examine the hypothesized relationships among career indecision, irrational beliefs, academic self-efficacy, and academic burnout. The subsequent qualitative phase was conducted to further interpret the quantitative findings and explore students' career development and academic experiences in greater depth.

### Participants and procedure

Ethical approval was obtained from the corresponding author's institution (Approval No. SFL2025006) before data collection. Participation was voluntary, and participants were informed that they could decline to answer any question or withdraw from the study at any time without penalty. Written informed consent was obtained from the participants' parents or legal guardians, while the participants themselves provided informed assent. All data were anonymized and used solely for research purposes.

Participants were recruited from four secondary vocational schools in the Tibet Autonomous Region and the Guangxi Zhuang Autonomous Region, China, including two schools in Tibet and two in Guangxi. Cluster sampling was conducted at the class level to facilitate group administration of the questionnaire. The target sample size was determined through an a priori power analysis using G^*^Power 3.1. Based on a significance level of α = 0.05, a statistical power of.95, and an anticipated effect size of *f* ^2^ = 0.11, the analysis indicated that at least 156 participants were required. Finally, 436 questionnaires were distributed. After patterned and incomplete responses were excluded, 398 valid questionnaires remained, yielding a valid response rate of 91.3%.

The final quantitative sample consisted of 398 students aged 15–18 years. Of these participants, 198 (49.7%) were male and 200 (50.3%) were female. The sample comprised 284 (71.4%) Zhuang students, 107 (26.9%) Tibetan students, 2 (0.6%) Yao students, and 5 (1.3%) students from other ethnic groups (including Menba and Lhoba). Regarding grade level, 229 (57.5%) were in Grade 1, 103 (25.9%) were in Grade 2, and 66 (16.6%) were in Grade 3. Participants studied in nine major categories, including Robotics/Mechatronics (*n* = 86, 21.6%), Computer and Information Technology (*n* = 75, 18.8%), Early Childhood Education (*n* = 66, 16.6%), Tourism and Hospitality (*n* = 81, 20.3%), E-commerce (*n* = 33, 8.3%), Tea Production and Processing (*n* = 33, 8.3%), Beauty and Hairdressing (*n* = 18, 4.5%), and Automotive and Garment (*n* = 6, 1.5%). In addition, 313 (78.6%) participants came from rural areas and 85 (21.4%) came from urban areas.

Following the quantitative survey, 10 participants were purposively selected for semi-structured interviews based on their questionnaire responses. Specifically, students were first grouped according to their questionnaire responses and academic performance. Participants representing relatively high, moderate, and low levels of career indecision, academic self-efficacy, and academic burnout were then invited to participate in the interviews to ensure variation in educational experiences and psychological characteristics. The demographic characteristics of the 10 interview participants were presented in Table 1.

**Table 1:** Demographic of qualitative participants.

Participants	Age	Academic year	Gender	Ethnicity	Household registration	Major	Career plan
Student 1(S1)	16	2	Male	Tibetan	Rural	Tea	Military
Student 2(S2)	16	1	Female	Zhuang	Rural	E-commerce	Anchor
Student 3(S3)	16	2	Female	Zhuang	Rural	ECE	No
Student 4(S4)	17	2	Male	Tibetan	Rural	Tea	Truck Driver
Student 5(S5)	16	1	Male	Tibetan	Urban	Travel	No
Student 6(S6)	16	2	Female	Lhoba	Rural	Tea	No
Student 7(S7)	17	3	Female	Tibetan	Rural	ECE	No
Student 8(S8)	16	2	Female	Tibetan	Rural	Tea	Doctor
Student 9(S9)	16	2	Female	Lhoba	Rural	Art	Farming
Student 10(S10)	15	1	Male	Zhuang	Urban	E-commerce	Anchor

### Research instruments

The present study employed a structured questionnaire consisting of four sections corresponding to the study variables: career indecision, irrational beliefs, academic self-efficacy, and academic burnout. All questionnaires were administered in Chinese. For career indecision, the study used items based on the Chinese version of the Career Decision-Making Difficulties Questionnaire (CDDQ) validated by Creed and Yin (2006). Their study highlighted the importance of considering cultural differences in career decision-making, particularly the influence of collectivism, social conformity, and family expectations in the Chinese context. Academic burnout was assessed using items from the Adolescent Student Burnout Inventory developed and validated among Chinese students by Wu et al. (2010), which was specifically developed to measure learning burnout among students across different educational stages in China. Thus, these two measures provided established Chinese-contextualized foundations for the present study.

For the measures of irrational beliefs and academic self-efficacy, the original English-language instruments were translated into Chinese and adapted to the educational context of Chinese secondary vocational students. The corresponding and fourth authors jointly translated the measures, after which a group of local secondary vocational school teachers reviewed the Chinese versions for linguistic clarity and contextual appropriateness. Particular attention was paid to ensuring that the items were readily comprehensible to secondary vocational students while preserving the conceptual meaning of the original constructs. The translated measures were then administered in a pilot study before the main survey. The pilot-test results provided preliminary evidence of acceptable internal consistency and construct suitability, with Cronbach's α values of 0.719 for irrational beliefs and.878 for academic self-efficacy, and KMO values of 0.594 and 0.789, respectively. Bartlett's tests of sphericity were significant for both measures (*p* < 0.001), providing preliminary support for their suitability for factor analysis. These results supported the use of the translated measures in the subsequent main study. Across all four measures, item wording was retained or minimally adapted where necessary to reflect the educational and vocational experiences of the target population while preserving the original conceptual meaning. All items were rated on a five-point Likert scale ranging from 1 (“strongly disagree”) to 5 (“strongly agree”).

### Career indecision (CI)

Career indecision was measured using the Career Decision Difficulties Questionnaire (CDDQ) validated by Creed and Yin (2006). Five items were selected and adapted from the original questionnaire to assess students' uncertainty regarding future career planning (e.g., “I know what kind of job I can do in the future”). Higher scores indicated greater career indecision. Cronbach's α for the present sample was 0.846.

### Irrational beliefs (IB)

Irrational beliefs were assessed based on the Irrational Beliefs Scale (IBS) originally developed by Malouff et al. (1987). Hyland et al. (2013) further examined the structural validity of the scale using confirmatory factor analysis and bifactor modeling, subsequently developing a shorter 24-item version with strong psychometric properties and satisfactory structural validity. Based on this revised version, three items were selected and adapted to reflect irrational learning- and career-related beliefs among ethnic minority secondary vocational students. Higher scores indicated stronger irrational beliefs. Cronbach's α for the present sample was 0.859.

### Academic self-efficacy (AS)

Academic self-efficacy was measured based on the core constructs of the Academic Self-efficacy Scale developed and validated by Chemers et al. (2001). Considering the educational and occupational characteristics of ethnic minority secondary vocational students, four representative items were retained and slightly reworded (e.g., “I believe I can master the core skills of my major” and “I believe I can acquire the skills required for employment in ethnic minority regions”). Higher scores indicated greater academic self-efficacy. Cronbach's α for the present sample was 0.880.

### Academic burnout (AB)

Academic burnout was assessed using selected items from the Adolescent Student Burnout Inventory developed by Wu et al. (2010). In this study, four representative items were selected and adapted to assess students' emotional exhaustion, learning disengagement, and academic fatigue. Higher scores indicated greater academic burnout. Higher scores indicated greater academic burnout. Cronbach's α for the present sample was 0.881.

### Interview protocol

The interview protocol was developed based on the theoretical framework of the study, integrating career decision-making difficulties theory, irrational beliefs theory, self-efficacy theory, and academic burnout models (Gati et al., 1996; Ellis, 1991; Bandura, 1977). The protocol focused on four areas: (1) career planning experiences, (2) beliefs regarding education and occupations, (3) perceptions of academic competence, and (4) experiences of academic disengagement or burnout. The interview guide was designed to explain and elaborate on the quantitative findings rather than to test new hypotheses, consistent with the sequential explanatory mixed-methods design. Example questions included: “How confident do you feel about completing difficult academic tasks, and what affects this confidence?”; and “Can you describe any experiences in which you felt exhausted, disengaged, or unmotivated in relation to your studies?”

Interviews were conducted individually in a quiet setting and lasted approximately 30–40 min. With participants' consent, all interviews were audio-recorded and subsequently transcribed verbatim. During the interviews, additional probing questions were used when necessary to encourage participants to elaborate on their responses.

### Data analysis

After data collection, the analyses were conducted in several stages. First, SPSS 27.0 was used to calculate descriptive statistics, including means and standard deviations, for all four study variables, and Pearson correlation analyses were subsequently performed to examine the relationships among these variables. Second, the PROCESS macro was employed to conduct mediation analyses related to Structural Equation Modeling (SEM) in order to determine whether the hypothesized chain mediation model presented in Figure 1 was supported. Finally, to evaluate the statistical significance of the mediation effects, a bootstrap procedure with 5,000 resamples was conducted to estimate confidence intervals for the indirect effects.

Qualitative data were analyzed using thematic analysis, supported by the constant comparative method (Braun and Clarke, 2006; Corbin and Strauss, 2014). Interview transcripts were first read repeatedly to achieve familiarity with the data and to identify recurring concepts and patterns. Preliminary coding was assisted by an AI-based analytical tool, while all coding decisions and thematic interpretations were independently reviewed and refined by the researchers. Similar codes were subsequently grouped into broader categories through repeated comparison across transcripts. Potential themes were then identified, reviewed, and refined iteratively to ensure internal coherence and clear distinctions between themes. The analysis ultimately generated four overarching themes related to career aspirations, irrational beliefs, academic self-efficacy, and academic burnout among ethnic minority secondary vocational students (Patton, 2014).

### Reliability and validity

The internal consistency reliability of all scales was assessed using Cronbach's α coefficient. The results indicated that all constructs demonstrated acceptable to good reliability, with Cronbach's α values ranging from 0.846 to 0.881. The KMO measures of sampling adequacy for all scales were above 0.5 (CI = 0.820, IB = 0.651, AS = 0.829, AB = 0.827), and Bartlett's tests were significant (*p* < 0.001), confirming the data's suitability for factor analysis. Principal component analysis showed that the communalities for all items were above 0.44, with most exceeding 0.65, supporting the scales' structural validity.

For the qualitative component, trustworthiness was established following the criteria proposed by Patton (Patton, 2014) and Corbin and Strauss (Corbin and Strauss, 2014). Credibility was enhanced through purposive maximum-variation sampling, which included both high-achieving and low-achieving ethnic minority vocational students, allowing diverse perspectives on career planning and learning experiences to be captured. During interviews, probing and follow-up questions were used to clarify participants' responses and reduce misinterpretation.

Transferability was supported through rich descriptions of participants' educational backgrounds, minority contexts, and vocational learning experiences, enabling readers to assess the applicability of the findings to similar educational settings.

Dependability was strengthened by maintaining a detailed audit trail throughout the research process, including interview protocols, transcription procedures, coding decisions, and theme development. Interview transcripts were repeatedly reviewed and compared using the constant comparative method to ensure consistency in theme generation.

Confirmability was enhanced by grounding interpretations in participants' verbatim quotations and by systematically comparing emerging themes against the original interview transcripts. AI-assisted coding was used only to generate preliminary codes and thematic patterns; all themes were subsequently reviewed, refined, and verified by the researchers to ensure that the findings accurately reflected participants' perspectives rather than researcher assumptions.

## Results

### Quantitative results

#### Common method bias

To examine the potential influence of common method bias, Harman's single-factor test was conducted using an unrotated exploratory factor analysis. The results showed that four factors with eigenvalues greater than 1 were extracted, and the first unrotated factor accounted for 30.79% of the total variance, which was below the commonly accepted threshold of 40%. Therefore, common method bias was unlikely to pose a serious threat to the findings of the present study.

#### Descriptive statistics

Descriptive statistics for the study variables were presented in Table 2. The mean scores for career indecision (M = 2.89, SD = 0.66), irrational beliefs (M = 2.78, SD = 0.70), academic self-efficacy (M = 3.10, SD = 0.68), and academic burnout (M = 2.88, SD = 0.68) indicated moderate levels across the four study variables. Academic self-efficacy showed a relatively higher mean level than the other variables, whereas irrational beliefs had the lowest mean score. The standard deviations ranged from 0.66 to 0.70, indicating comparable variability across the study variables.

**Table 2:** Descriptive statistics and correlations.

Variables	M	SD	1	2	3	4
1 CI	2.89	0.66	1			
2 IB	2.78	0.70	0.361^**^	1		
3 AS	3.10	0.68	−0.358^**^	−0.052	1	
4 AB	2.88	0.68	0.177^**^	0.222^**^	−0.282^**^	1

^**^ *p* < 0.01.

#### Correlation analysis

Pearson correlation analysis was conducted to examine the relationships among the four major variables, and the results were presented in Table 2.

Career indecision (CI) was significantly positively correlated with irrational beliefs (IB) (*r* = 0.361, *p* < 0.01) and academic burnout (AB) (*r* = 0.177, *p* < 0.01), while it was significantly negatively correlated with academic self-efficacy (AS) (r = −0.358, *p* < 0.01). Irrational beliefs (IB) showed a non-significant negative correlation with academic self-efficacy (AS) (*r* = −0.052). However, IB was significantly positively correlated with academic burnout (AB) (*r* = 0.222, *p* < 0.01). Academic self-efficacy (AS) was significantly negatively correlated with academic burnout (AB) (*r* = −0.282, *p* < 0.01). Overall, most correlations among the study variables reached statistical significance at the 0.01 level.

#### Mediation model results

**Figure 2:**
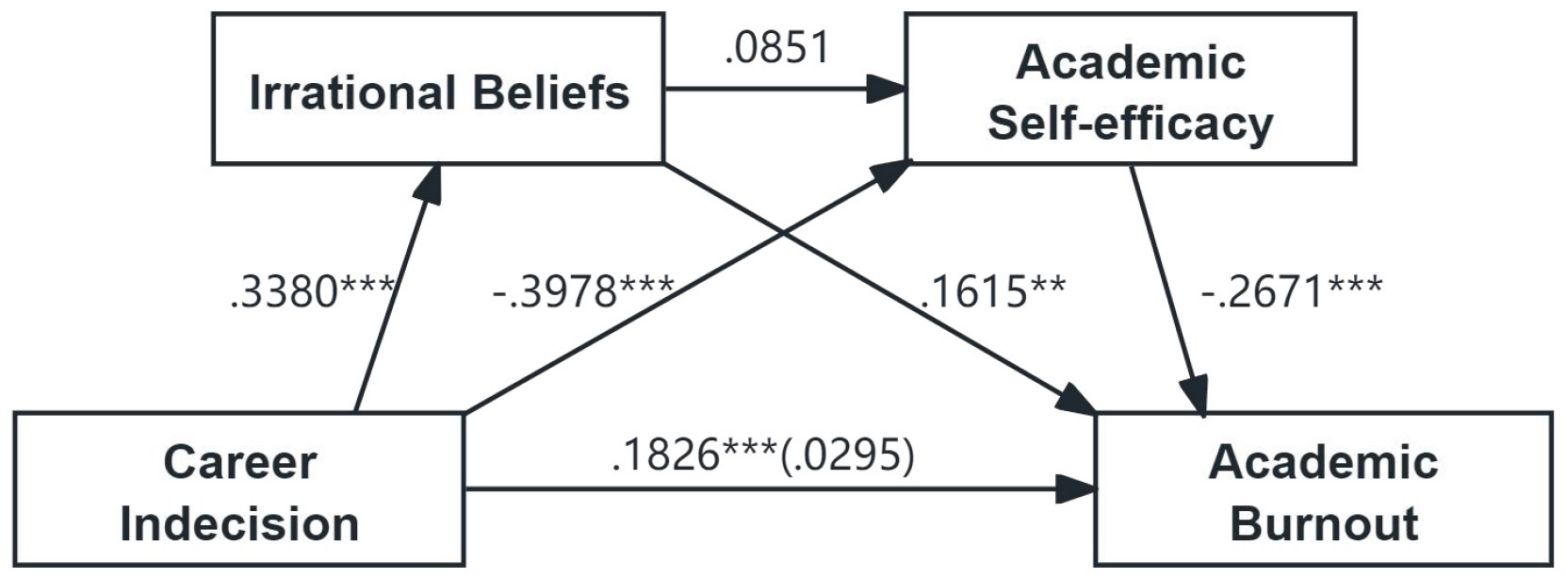
Chain mediation model diagram. ** *p* < 0.01, *** *p* < 0.001.

To examine the mechanisms underlying the relationship between career indecision (CI) and academic burnout (AB), a chain multiple mediation model (Model 6) was tested using the SPSS PROCESS macro, with irrational beliefs (IB) and academic self-efficacy (AS) as mediators. The hypothesized mediation model and the relationships among the variables were presented in Figure 2.

#### Total and direct effects

As shown in Table 3, the total effect of CI on AB was significant and positive (effect = 0.1826, SE = 0.0512, *t* = 3.5691, *p* = 0.0004). However, after including both mediators, irrational beliefs and academic self-efficacy, in the model, the direct effect of CI on AB became non-significant (effect = 0.0295, SE = 0.0558, *t* = 0.5281, *p* = 0.5977).

**Table 3:** Effects of the mediation model.

Effect type	Path	Effect	SE	95% CI
Total effect	CI → AB	0.1826	0.0512	[0.0820, 0.2832]
Direct effect	CI → AB	0.0295	0.0558	[−0.0802, 0.1391]

#### Path coefficients

The regression analyses for the mediation model revealed several significant pathways, as presented in Table 4. First, CI significantly and positively predicted IB (coeff = 0.3380, SE = 0.0501, *t* = 6.7438, *p* < 0.001). Second, CI significantly and negatively predicted AS (coeff = −0.3978, SE = 0.0509, *t* = −7.8217, *p* < 0.001). However, the path from IB to AS was not statistically significant (coeff = 0.0851, SE = 0.0483, *t* = 1.7629, *p* = 0.0787).

**Table 4:** Results of chain mediation analysis using PROCESS model 6 regression paths.

Path	Outcome	Predictor	B	β	SE	*t*	*R* ^2^	*p*
M1	IB	CI	0.3380	0.3210	0.0501	6.7438	0.1030	0.0000
M2	AS	CI	−0.3978	−0.3864	0.0509	−7.8217	0.1353	0.0000
M2	AS	IB	0.0851	0.0871	0.0483	1.7629		0.0787
Y	AB	CI	0.0295	0.0285	0.0558	0.5281	0.1102	0.5977
Y	AB	IB	0.1615	0.1644	0.0495	3.2633		0.0012
Y	AB	AS	−0.2671	−0.2659	0.0513	−5.2021		0.0000

M1, first mediator; M2, second mediator; Y, dependent variable.

Regarding the dependent variable, both mediators significantly predicted AB. Specifically, IB positively predicted AB (coeff = 0.1615, SE = 0.0495, *t* = 3.2633, *p* = 0.0012), whereas AS negatively predicted AB (coeff = −0.2671, SE = 0.0513, *t* = −5.2021, *p* < 0.001).

#### Indirect effects

A bootstrapping procedure with 5,000 resamples was conducted to examine the significance of the indirect effect, as presented in Table 5. An indirect effect was considered statistically significant when the 95% bootstrap confidence interval (CI) did not include zero. The total indirect effect of CI on AB was significant (Effect = 0.1532, SE = 0.0501, 95% CI [0.0637, 0.2577]).

**Table 5:** Specific indirect effects.

Mediating path	Effect	SE	95% CI
CI → IB → AB (Ind1)	0.0546	0.0285	[0.0066, 0.1181]
CI → AS → AB (Ind2)	0.1063	0.0385	[0.0389, 0.1908]
CI → IB → AS → AB (Ind3)	−0.0077	0.0065	[−0.0220, 0.0041]

Bootstrap estimates were based on 5,000 resamples.

Three specific indirect pathways were further examined. Path 1 (Ind1: CI → IB → AB) was significant (effect = 0.0546, SE = 0.0285, 95% CI [0.0066, 0.1181]), indicating that higher CI was indirectly associated with higher AB through higher levels of irrational beliefs. Path 2 (Ind2: CI → AS → AB) was also significant (effect = 0.1063, SE = 0.0385, 95% CI [0.0389, 0.1908]), indicating that higher CI was indirectly associated with higher AB through lower academic self-efficacy. Path 3 (Ind3: CI → IB → AS → AB) was not significant because its 95% confidence interval included zero (effect = −0.0077, SE = 0.0065, 95% CI [−0.0220, 0.0041]).

### Qualitative results

Analysis of the interview data generated four overarching themes that corresponded to the four quantitative constructs examined in this study: career indecision, irrational beliefs, academic self-efficacy, and academic burnout. As part of the sequential explanatory mixed-methods design, the qualitative findings complemented the quantitative results by illustrating how these psychological experiences were reflected in students' educational and career-development experiences. The four themes were: (1) fragmented career aspirations and uncertainty about future planning, (2) irrational beliefs toward education and occupational development, (3) polarization of academic self-efficacy, and (4) academic burnout associated with major mismatch. These findings emerged within the contextual characteristics of ethnic minority vocational education, where students encountered distinctive challenges related to educational resources, career information access, family educational support, and opportunities for occupational exploration.

### Theme 1: fragmented career aspirations and uncertainty about future planning

A dominant theme emerging from the interviews was the fragmented and unstructured nature of students' career aspirations. Although several participants expressed interest in future occupations, most lacked specific implementation plans, knowledge regarding educational pathways, or alternative strategies. Career expectations often appeared to be based on vague personal preferences or environmental exposure rather than systematic career planning. Several participants indicated that their career expectations were shaped primarily by family experiences, local employment environments, or limited social exposure rather than systematic career exploration.

Many participants demonstrated uncertainty regarding how occupational goals could be translated into concrete actions. For example, one student expressed a desire to pursue a professional path but simultaneously admitted limited knowledge about the requirements needed to achieve it: “I want to become a doctor, but I haven't really figured it out yet. I don't know what steps I should take next. I just think becoming a doctor sounds good.” (S8)

Others reported complete absence of career goals, with statements such as “I haven't made any plans for the future yet” (S6) or “I don't want to do anything” (S5). This finding was consistent with the quantitative results showing that higher career indecision was significantly associated with higher academic burnout and lower academic self-efficacy.

### Theme 2: irrational beliefs toward education and occupational development

Another recurring pattern involved irrational beliefs regarding the value of education and future career development. Participants frequently questioned the usefulness of academic learning and viewed educational attainment primarily as a means of obtaining basic credentials rather than developing competencies or achieving long-term goals. Occupational choices were also often evaluated mainly through immediate economic returns rather than personal interests or developmental opportunities. Six participants evaluated education mainly through immediate practical benefits and employment outcomes, reflecting the relation of surrounding social experiences and limited awareness of diverse educational pathways. This perception was illustrated by S4 who repeatedly questioned the usefulness of schooling: “I don't think studying can provide me with the ability needed for future work. These courses are useless to me. Some people graduate from university and still come back without jobs. I can drive heavy trucks, as long as I get a diploma and a driver's license, that should be enough.” (S4) This theme corresponded to the quantitative finding that stronger irrational beliefs were associated with higher academic burnout.

### Theme 3: polarization of academic self-efficacy

Participants demonstrated substantial differences in academic self-efficacy, with responses generally falling into high- and low-efficacy patterns. Students with higher self-efficacy typically reported stronger confidence in their professional abilities and more proactive learning behaviors, whereas students with lower self-efficacy frequently doubted their academic capabilities and questioned the relevance of learning activities to future employment. This contrast was particularly evident in students who actively engaged in learning and expressed confidence in future development: “As the class monitor, I usually pay attention in class. I ranked first in English in my class, and I have confidence in myself. I also want to take the college entrance examination.” (S8)

In contrast, students with lower self-efficacy repeatedly emphasized feelings of incompetence and disengagement. This pattern supported the quantitative finding that academic self-efficacy was negatively associated with academic burnout.

### Theme 4: academic burnout associated with major mismatch

Academic burnout emerged as a common experience among participants and was frequently associated with involuntary major selection or mismatch between students' interests and their assigned fields of study. Many students reported that their current majors were not their first choices and had instead been selected randomly or assigned through administrative adjustments. This lack of personal choice appeared to reduce students' motivation and investment in learning activities. One participant explicitly stated: “Once you've already decided what you want to do in the future, you won't put that much effort into classes anymore.” (S4)

Overall, the findings provided partial support for the proposed hypotheses. H1 was supported, indicating that career indecision was significantly associated with academic burnout. H2 was also supported, as career indecision was positively associated with irrational beliefs, irrational beliefs were negatively associated with academic self-efficacy, and academic self-efficacy was negatively associated with academic burnout. Regarding H3, the parallel mediating effects of irrational beliefs and academic self-efficacy were supported, whereas the proposed sequential mediation pathway through irrational beliefs and academic self-efficacy was not supported. In addition, the qualitative findings were generally consistent with the quantitative results and reflected the major psychological patterns identified in the statistical analyses.

## Discussion

The present study aimed to clarify the psychological mechanisms through which career indecision (CI) was associated with academic burnout (AB) among ethnic minority secondary vocational students in China. Overall, the findings partially supported the proposed hypotheses. The results indicated that although career indecision was significantly associated with academic burnout at the total effect level, this relationship was fully transmitted through indirect pathways involving irrational beliefs (IB) and academic self-efficacy (AS). Specifically, career indecision significantly predicted irrational beliefs and academic self-efficacy while both irrational beliefs and academic self-efficacy were significantly associated with academic burnout, operated through internal cognitive and motivational mechanisms. However, the hypothesized chain mediation pathway through both IB and AS was not supported. These findings highlight how the relationship between career uncertainty and academic burnout may operate differently within the contextual environment of ethnic minority vocational education. Rather than reflecting a simple deficit in motivation or academic ability, students' burnout experiences appeared to emerge from the interaction between limited career development opportunities and individual psychological processes.

### The relationship between CI and AB

The present study found that career indecision was not directly associated with academic burnout after the mediating variables were included in the model. This finding differed from previous research (Kusumawardani and Hidayat, 2024), which reported a positive association between career indecisiveness and academic burnout among Indonesian adolescents. There were two possible explanations for this discrepancy, primarily related to contextual factors and student resilience.

One possible explanation was that contextual and cultural environments shaped students' value systems differently (Yang et al., 2022). Qualitative findings also indicated that some students maintained relatively high levels of learning engagement despite expressing uncertainty about future occupational directions. Previous research suggested that students in ethnic minority regions might have relatively limited access to career information, occupational networks, and career guidance resources, which might constrain systematic career exploration (Wang, 2023). Under such circumstances, career uncertainty might not necessarily translate directly into academic burnout, as students often continued to engage in learning despite unclear future plans.

Another possible explanation related to the resilience and adaptive characteristics commonly observed among students in ethnic minority regions. Occupational uncertainty might coexist with relatively stable academic engagement because students often derived meaning from broader sources, including family expectations, practical skills, and community roles (Doan et al., 2022; Hill, 2022). Therefore, career indecision alone might not be sufficient to generate burnout symptoms.

The full mediation pattern suggested that career indecision itself might not be sufficient to be associated with academic burnout. Instead, its association appeared to depend on how students cognitively interpreted career uncertainty and evaluated their own academic competence. Students who experienced career uncertainty might not necessarily develop burnout unless such uncertainty was accompanied by maladaptive beliefs or diminished confidence in their academic abilities. Therefore, irrational beliefs and academic self-efficacy appeared to represent two key psychological mechanisms linking career indecision with academic burnout in this population. Although the direct pathway was non-significant, the overall indirect effect remained significant. This finding suggested that the relation of career indecision on academic burnout might be psychologically complex and primarily operated through internal cognitive and motivational mechanisms rather than through a direct pathway.

### The relationship between CI and IB

The present study confirmed a significant positive relationship between career indecision and irrational beliefs, supporting Hypothesis H2a. This finding was consistent with previous studies suggesting that irrational beliefs regarding occupational choices were associated with career indecisiveness rather than career maturity (Hamamci and Esen Çoban, 2007). Similarly, previous research reported that adolescents experiencing difficulties in career planning tended to exhibit stronger irrational career beliefs (Ari and Eşkisu, 2025), while parental expectations and criticism were positively associated with both career decision-making difficulties and maladaptive occupational beliefs.

The present findings further reflected the contextual characteristics of students in ethnic minority regions. The qualitative interviews suggested that many participants evaluated education primarily in terms of immediate employment opportunities and practical skills while questioning the long-term value of academic learning. Similar observations were reported among students in ethnic minority regions, where educational decisions were often influenced by local employment contexts and relatively limited awareness of diverse educational and occupational pathways (Bolan and Mingxing, 2023). Such contextual experiences might contribute to the development of irrational beliefs regarding education and career development.

Previous studies indicated that parenting styles shaped personality development and relates to career decision-making processes (Kiff et al., 2011; Ng and Wei, 2020). Such contextual experiences might be an important correlation of the development of rigid expectations, negative assumptions, or maladaptive beliefs regarding future occupational choices. These findings suggested that uncertainty regarding future career directions might increase the likelihood of maladaptive cognitive interpretations, thereby reinforcing irrational beliefs among vocational students.

### The relationship between AS and AB

The findings showed that academic self-efficacy was significantly negatively associated with academic burnout, supporting Hypothesis H2c. This result was consistent with previous studies demonstrating a close relationship between self-efficacy and academic burnout across different educational settings (Kong et al., 2021; Ma, 2024).

Previous research among Chinese EFL learners reported that academic self-efficacy and academic motivation strongly predicted academic burnout (Ma, 2024). Similar findings among nursing students further indicated that proactive personality traits and occupational self-efficacy were negatively associated with burnout symptoms (Kong et al., 2021). The present study extended these findings to secondary vocational students from ethnic minority regions and further confirmed the robustness of this relationship.

According to self-efficacy theory (Bandura, 1977), students who perceived themselves as capable of handling academic demands were more likely to maintain motivation, persistence, and adaptive coping behaviors when encountering learning challenges. Conversely, students with lower confidence in their academic abilities might gradually disengage from learning activities, ultimately increasing their vulnerability to burnout symptoms (Schunk, 1995). This mechanism might be particularly relevant among ethnic minority vocational students. Qualitative findings revealed substantial differences in students' confidence in learning, with some participants actively seeking academic support while others repeatedly expressed feelings of incompetence and disengagement. These differences might partly reflect disparities in prior educational experiences, family educational support, and access to learning resources before entering vocational schools (Doan et al., 2022; Guo et al., 2020). Such contextual factors might shape students' perceptions of academic competence and their vulnerability to academic burnout.

### The chain mediation mechanism of IB and AS

The hypothesized relationship between irrational beliefs and academic self-efficacy was not supported. This finding differed from previous studies suggesting that irrational beliefs undermined self-efficacy through distorted interpretations of challenges and failures (Ozer and Akgun, 2015; Kabasakal and Emiroglu, 2020; Gkontelos et al., 2023; Coşa and Cernat, 2025). Several explanations might account for this discrepancy.

First, among students in ethnic minority vocational schools, self-worth systems might be relatively independent from academic achievement. Within mainstream educational contexts, perceptions of competence were often strongly linked to academic performance and educational opportunities (Chamberlin et al., 2023). In contrast, vocational students in ethnic minority regions derived self-worth more strongly from family responsibility, community recognition, traditional skills, and practical competence (Yang et al., 2019). Findings from the qualitative research suggested that many students placed greater value on acquiring practical qualifications, occupational skills, and future employability than on academic performance itself. This pattern was also consistent with previous evidence suggesting that practical employment opportunities were often prioritized over long-term educational development in some ethnic minority contexts (Bolan and Mingxing, 2023), which might weaken the association between irrational beliefs and academic self-efficacy. Consequently, irrational career-related beliefs might not be directly associated with their evaluations of academic capability.

Second, findings from this study indicated a perceived mismatch between academic learning and future occupational development. Some students reported limited confidence in academic subjects while simultaneously expressing confidence in their practical abilities and future employment prospects. This pattern suggested that academic competence and occupational competence might be viewed as relatively independent domains. Under such circumstances, irrational beliefs related to career planning might not necessarily translate into lower levels of academic self-efficacy.

Taken together, these findings suggested that irrational beliefs and academic self-evaluations might function as relatively independent psychological dimensions in this population, which might explain the non-significant relationship between IB and AS and the absence of the hypothesized chain mediation pathway. This finding suggested that ABC theory and self-efficacy theory might operate in parallel rather than sequentially in explaining academic burnout among ethnic minority vocational students. Therefore, interventions targeting maladaptive beliefs and strengthening academic self-efficacy might represent complementary rather than hierarchical approaches.

### Implications and conclusion

The present study had the potential to contribute to the theoretical understanding of academic burnout among ethnic minority secondary vocational students in several ways. First, by integrating ABC theory and self-efficacy theory, the findings suggested that these two theoretical perspectives might complement each other in explaining how career-related cognitive processes were associated with academic burnout. Specifically, the results suggested that irrational beliefs and academic self-efficacy might represent important psychological mechanisms through which career indecision was related to students' academic experiences. This integrated perspective might broaden the application of these theories from general educational and emotional adjustment to career-development contexts within vocational education. Second, although the proposed sequential mediation pathway was not supported, the significant parallel mediating roles of irrational beliefs and academic self-efficacy suggested that multiple psychological processes might operate simultaneously rather than sequentially in understanding academic burnout. These findings provided a nuanced perspective on the psychological mechanisms underlying career indecision and might inform future theoretical refinement of mediation models in educational psychology. Finally, by employing a sequential explanatory mixed-methods design, the present study illustrated how quantitative and qualitative evidence might be integrated to provide complementary perspectives on students' psychological experiences, thereby offering a methodological reference for future research involving underrepresented educational populations.

By validating this full mediation model, the present study advanced the question of whether career indecision and academic burnout were related toward a deeper understanding of how this relationship operates psychologically. Practically, the findings suggested that interventions should not merely focus on reducing occupational uncertainty itself, but should instead prioritize correcting irrational beliefs and cultivating resilient academic self-efficacy in order to interrupt the maladaptive transmission process related to burnout.

The findings further suggested that schools should strengthen the dissemination of career planning education and provided more targeted psychological counseling services (Liu et al., 2023; Zhang et al., 2025). Through occupational information guidance and interest-oriented career cultivation, educators might gradually reduce students' perceptions that vocational education equated to “having no future”, thereby promoting diversified development opportunities and potentially improving students' long-term academic and psychological outcomes.

This study had limitations. First, due to practical constraints preventing long-term residence in ethnic minority regions, the study adopted a cross-sectional design rather than employing Dynamic Structural Equation Modeling to observe longitudinal changes in students' psychological states. Consequently, the study was unable to explore developmental changes or intervention effects over time.

Second, the representativeness of the sample remained limited. The study covered four schools across two ethnic minority provinces, but broader ethnic diversity and regional variation were not fully represented. Future research should therefore expand sampling across multiple ethnic groups and regions while simultaneously examining whether long-term career planning interventions and psychological counseling programs may alter the mediation pathways over time.

Future studies may employ longitudinal or intervention-based designs to examine whether improving career planning, correcting irrational beliefs, and strengthening academic self-efficacy could reduce academic burnout over time. In addition, future research could compare different ethnic groups and educational settings to determine whether the present findings are generalizable across diverse cultural contexts.

The present study demonstrated that the association of career indecision on academic burnout was primarily transmitted through indirect psychological pathways rather than through a direct effect. In this population, career indecision did not automatically translate into burnout. Instead, its relation depended on how students interpret educational value and evaluate their academic competence within a specific vocational and regional context. The findings underscored the importance of cognitive beliefs and academic self-perceptions in shaping students' academic experiences. By focusing on secondary vocational students in ethnic minority regions, this study extended existing knowledge beyond mainstream educational contexts by demonstrating how career uncertainty may interact with contextual factors, such as limited career exploration opportunities, educational experiences, and local employment environments, to contribute to academic burnout.

## Data Availability

The raw data supporting the conclusions of this article will be made available by the authors, without undue reservation.
